# Reliability Study of a Functional Test for the Offensive Agility Performance in Water Polo

**DOI:** 10.3390/ijerph191610040

**Published:** 2022-08-15

**Authors:** Marcell Fridvalszki, János Matlák, Bálint Kovács, Leonidas Petridis, Dávid Horváth, Krisztián Havanecz, Donatella Dudás, Gergely Langmár, Levente Rácz

**Affiliations:** 1 Department of Kinesiology, Hungarian University of Sports Science, 1123 Budapest, Hungary; 2 Research Center for Sport Physiology, Hungarian University of Sports Science, 1123 Budapest, Hungary; 3 Department of Training Theory and Methodology Research Center, Hungarian University of Sports Science, 1123 Budapest, Hungary; 4 Ferenvárosi Torna Club Waterpolo Ltd., 1091 Budapest, Hungary; 5 Sport Science and Sport Health Department, Hungarian National Academy of Handball, 8630 Balatonboglár, Hungary

**Keywords:** open skill, decision-making, shooting efficiency

## Abstract

The aim of the study was to develop and assess the reliability of a functional agility test containing offensive elements for water polo players. Eighteen young male (15.3 ± 0.5 years, 178.3 ± 4.7 cm, 69.4 ± 10.0 kg) water polo players with a minimum of 5 years of sport-specific experience participated in this study. The test contained reactive high-intensity short-term swimming with changes in direction and manoeuvres after perceiving unknown stimuli given by tester players, and also included a shooting task at a goal, first from 7 m and then from 5 m. Execution time and shooting efficiency were measured by two experienced water polo coaches (Evaluators A and B). All statistical analyses were calculated using SPSS. The intrarater reliability between attempts showed good reliability for both evaluators (Evaluator A: ICC: 0.87; 95% CI: 0.66–0.95 and Evaluator B: ICC: 0.88; 95% CI: 0.68–0.96). Interrater reliability between Evaluators A and B was excellent at both attempts (Attempt 1: ICC: 0.97; 95% CI: 0.93–0.99 and Attempt 2: ICC: 0.98; 95% CI: 0.91–0.99). A lack of correlation between shooting performance from 7 m and 5 m distances and execution time was observed in the protocol. The test we presented in this study was found to be a reliable measurement tool for testing offensive agility performance based on open skill nature among water polo players.

## 1. Introduction

Water polo is an open skill team sport where the athletes are required to perform quick movements and positional and directional changes based on the observations of the ball, their opponents, teammates and the referee [1]. Therefore, water polo players need to have a high level of agility, which has been defined as “a rapid whole-body movement with change of velocity or direction in response to a stimulus” [2].

In team sports, there is an increasing number of researchers who have noticed the importance of decision-making and perceptual elements in agility measurements [2,3,4,5,6,7,8,9]. Furthermore, it was suggested that more sport-specific agility testing protocols should be created that can mimic real game situations [10]. Testing protocols in water polo, however, include mostly closed skills tasks [11,12,13], without considering the importance of the perceptive and cognitive capabilities that play a determinant role in water polo performance [14]. In the literature, few researchers have focused on the measurements in which the task is based on the open skill nature [1,14,15,16,17], where the response to a sensory stimuli is not automated or rehearsed [2].

In an earlier study, Falk et al. [18] compared motor ability, technical skills and game intelligence between selected and non-selected water polo players. They observed differences between selected players (into the national team) and non-selected players in motor abilities and in game intelligence, but not in a goal shooting task after a 2-year follow-up. This lack of difference was explained by the way the task was performed using only closed skills (without a goalkeeper and from a stationary position), which can influence shooting performance. The authors recommended including ‘game-like’ elements that possibly require anticipating and focusing attention on future tests, since under competitive conditions the selected players presumably can perform more efficiently.

More recent studies have aimed to develop sport-specific agility tests, which also include cognitive elements, such as decision making. For example, Tucher et al. [15] presented a sport-specific agility test to evaluate water polo players. The authors concluded that the test was more applicable for testing defensive actions than offensive actions since dribbling or throwing at a goal were not among the tasks. Moreover, in a later study, they suggested that the characteristics of the offensive actions should be included in future open tests [16]. Dong et al. [1] investigated the agility performance of water polo players, where the tasks were initiated by a light stimulus; however, this study represented only general aspects of agility and contained no defensive or offensive elements.

Currently, these tests [1,14,15,16,17] are referred to as sport-specific in-water agility, but none of these protocols focus on the offensive components of water polo gameplay. Based on the recent literature, offensive-style agility tests performed in water polo are not a well-studied topic, unlike in other team sports, where several tests have been developed focusing on the attacking part of the gameplay [19,20]. In studies with Australian football players [21,22] the authors showed a clear difference between offensive and defensive agility and suggested training and testing the athletes according to the role of the player. Young and Murray [21] noted that offensive and defensive agility have different qualities in physical, technical and cognitive demands; thus, more sport-specific offensive agility tests need to be developed.

Falk et al. [18] have proposed that an offensive agility test should include high-intensity starts from one place to another, manoeuvres after perception and anticipation of the opponent’s unexpected external cues followed by a shooting accuracy exercise, since this was suggested to be a major requirement in the gameplay of water polo. The purpose of this study was to develop a water-polo specific agility test focusing on offensive qualities and to test its reliability between repetitive attempts and between evaluators. The functional test consists of rapid changes in direction, changes between horizontal and vertical body positions, presenting an open skill nature and also include a shooting task at a goal.

## 2. Materials and Methods

### 2.1. Participants

Following a convenience sampling method, players, who fulfilled the inclusion criteria participated in the study. Eighteen young male competitive water polo players (age: 15.3 ± 0.5 years, body height: 1.78 ± 0.05 m, body weight: 69.4 ± 10.0 kg) were included in this study (3 centers, 4 guards, 5 wings, 5 drivers, 1 goalkeeper). The participants were played in the national age-group league. A minimum of five years of sport-specific experience was an inclusion criterion. The participants were healthy and had no musculoskeletal injury or pain in the lower or upper extremities at the time of the measurements.

### 2.2. Measure

**Figure 1:**
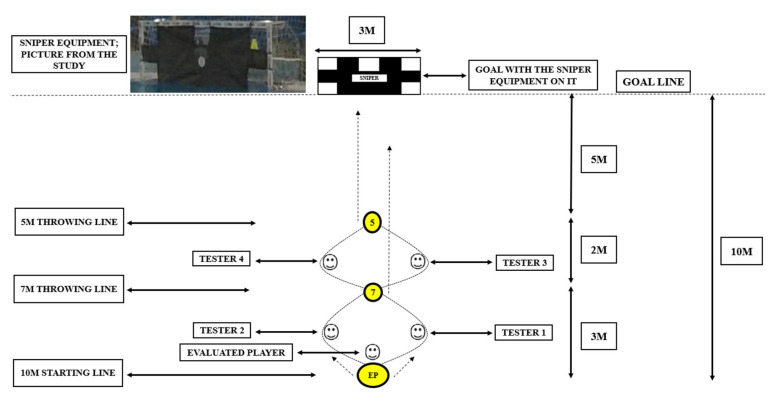
Schematic representation of the Functional Test for Offensive Agility Performance proposed to evaluate water polo players.

Measurements were performed in November, during the first half of the competitive period in a 10 m long, 3 m wide area marked in an official competition swimming pool. In this area, there were three swim-off floating rigs (10 m, 7 m and 5 m apart from the water polo goal) with a water polo ball inside (Figure 1). To ensure that the balls were not able to move away (from this spot) during the executions, these rigs were stabilized at the starting point (10 m), at the first throwing point (7 m) and at the second throwing point (5 m) with a 5 kg rubber-proofed disc weighted to the bottom of the pool.

Based on the studies of Royal et al. [23] and Stevens et al. [24], the shooting accuracy was measured via a designated cutout area and a shooting sieve through the goal called ‘Sniper’. With this equipment, we could ensure standardization of the shooting efficiency task; this is an accepted method to determine the players’ shooting performance. This equipment is a mesh sieve that completely fits the face of the water polo goal. It contains five 1 × 1-ft square cutouts, one in each corner of the goal and one in the upper middle (Figure 1).

Youth water polo players with more than 10 years of playing experience participated in the study (to show the signals for the players) as testers (T1–T4). They were positioned in a square pattern (pairs in two rows). In the first row, T1 and T2 were 8.5 m from the goal, whereas the second row, with T3 and T4, was positioned at 6 m. In both rows, the distance between them was 3 m. Special buoyancies on the dryland were used to ensure that T1–T4 stayed in the predetermined, fixed position during the test. Before every execution, the measuring team checked that the positions of T1–T4, the player being evaluated (EP) and the floating rigs were in the proper positions. The locations of the equipment and players with the tester are shown in Figure 1.

### 2.3. Procedure of the Functional Test for Offensive Agility

Before the testing day, there was a meeting day with every EP when the protocol was introduced, several videos about the task were presented to them, and the definition of the open skill and the requirements of agility movement were clarified. Additionally, every EP and their coaches were informed not to train 24 h prior to the testing day, and furthermore, they were told that a meal should be eaten at least 90 min before the commencement of the test.

Despite this meeting day’s occasion before completing the test, these videos were shown again, and ad hoc questions about the protocol were raised by the measuring team to ensure that the procedures were clear and verbal familiarization was given. Since the key point of the study was to maintain the open skill nature, not only was the protocol unknown for the EP, but the dressing room (which was closed to the civil population to ensure that the testing protocols were not disturbed) was separated into two parts (entering into the pool and exiting after execution).

The participants were tested in 3 groups. Furthermore, to maintain the unpredictable characteristics of the given movements, a one-hour break was ensured for the testers (T1–T4) outside the pool.

During the test, the EP’s objective was to swim and manoeuvre within the shortest possible time after the perception of the testers’ external stimuli, whereas T1–T4 had to maintain the high level of focus to create a ‘gameplay’ nature with the unexpected straight arm move.

The standardized 5-min-long dryland warm-up was followed by a 15 min in-water warm-up part with three blocks. The blocks consisted of swimming drills, ball technical drills and shot attempts. The warm-up parts were held by certified coaches. The continuity of warm-up protocols was strictly supervised by a dedicated staff member ensuring the test was performed no more than 5 min after the in-water warm-up.

After the warm-up, the EP was in a vertical position facing in the opposite direction to the test court and had one hand on the ball (10 m), which was in a swim-off floating rig near him; this was considered the starting point (Figure 1). The test began when the EP removed his hand from the starting point ball (10 m) and turned in front of the court as fast as possible. When T1 and T2 realized this movement based on the predetermined plan (unknown to the EP), one tester raised one of his arms straight up in the air as fast as possible. Perceiving this, the EP had to swim as fast as possible to the 7 m point by passing around the tester who did not move. After passing around, the EP had to grab the water polo ball from the swim-off floating rig and throw it at the goal immediately through the equipment discussed above. Then, T3 and T4 performed the same predetermined signal as T1 and T2 before. After the EP realized the signal, he had to swim as fast as possible to the 5 m floating rig point by passing around the Tester, who did not move, grab the ball and throw it at the goal immediately. The measurement was stopped when the ball left the hand of the EP.

The protocol was repeated 5 times, and a 3-min resting period was allowed between each repetition. Every participant had 3 practice trials and 2 timed trials that were later statistically analysed. It is important to note that if the performance was hindered by a human mistake (e.g., the ball slipped out from the hand), the test was repeated after the next EP completed his test.

Tests were manually measured by using two chronometer professional stopwatches (CHRO108, Tremblay, Gleizé, France). The measurements were measured by two highly experienced water polo coaches (Evaluators A and B). Both evaluators were completely involved in the protocol’s procedure and were verbally familiarized with the test. To avoid interference, Evaluators A and B were not allowed to talk to each other or get into conversations with other staff members during the measurements. Furthermore, the evaluated players did not receive any details about their time results.

### 2.4. Statistical Analysis

All statistical analyses were calculated using SPSS 25.0 (SPSS Inc., Chicago, IL, USA). To determine the relative between-evaluator reliability of attempts, an intraclass correlation coefficient (ICC) was calculated using a two-way mixed-effects model (average measures), along with the upper and lower 95% confidence intervals (CI). We considered the evaluators as a fixed effect and the participants as a random effect. The variable used in reliability calculations was the execution time to complete the functional test. The ICC estimate was considered good between 0.75 and 0.9 and excellent above 0.9 [25]. A Bland–Altman plot was used to determine the bias between the evaluators and the limits of agreement. Spearman rank correlation was used to determine the magnitude and direction of the correlation between the time results of the agility test and the succession of the attempted shots from 5 m and 7 m. Shooting accuracy between 5 m and 7 m lines was compared using chi-square test with odds ratios. In all cases, an alpha level of <0.05 was considered statistically significant.

## 3. Results

### 3.1. Reliability Results

The difference between Evaluators A and B for the first attempt was 0.05 ± 0.33 s and for the second attempt, 0.07 ± 0.27 s (Table 1). The difference between Attempt 1 and 2 was 0.72 ± 0.64 s for Evaluator A and 0.74 ± 0.64 s for Evaluator B. The mean coefficient of variation (CV) for each player between attempts was 4.32 ± 3.27% for Evaluator A and 4.68 ± 3.14% for Evaluator B. Between evaluators, the CV for each player was 0.30 ± 1.20% for Attempt 1 and 0.40 ± 1.33% for Attempt 2. The players’ performance showed moderate variability between the two attempts, which explains the difference in execution time; however, the difference between Evaluators A and B was small at both measurements (on average 0.06 ± 0.30 s).

**Figure 2:**
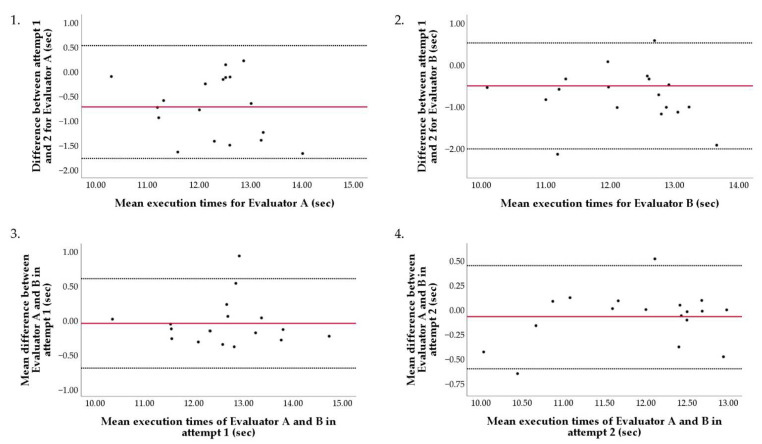
Bland–Altman plots of the measured execution times between Evaluator A and B, and between Attempts 1 and 2. Comparisons are illustrated by differences between pairs of measurements as a function of the mean measurements. Panel **1** shows the comparison between Attempts 1 and 2 for Evaluator A, and Panel **2** for Evaluator B. Panel **3** shows the comparison between Evaluator A and Evaluator B for Attempt 1 and Panel **4** for Attempt 2. Red line represents the mean difference between the corresponding compared measurements. The dotted black lines demonstrate the upper and lower bound of the 95% confidence intervals.

The reliability test results showed good intrarater reliability between Attempts 1 and 2 for Evaluators A (ICC: 0.87; 95% CI: 0.66–0.95) and Evaluator B (ICC: 0.88; 95% CI: 0.68–0.96) despite a marked difference in execution time between Attempts 1 (0.72 ± 0.64 s) and 2 (0.74 ± 0.64 s). Interrater reliability between Evaluators A and B was excellent in Attempt 1 (ICC: 0.97; 95% CI: 0.93–0.99) and Attempt 2 (ICC: 0.98; 95% CI: 0.91–0.99) (Figure 2).

### 3.2. Execution Time Descriptive Results

On average, mean execution time was 12.1 ± 0.9 s. The slowest time was 13.8 s, whereas the fastest time was 10.2 s. Using the mean ± 0.5 sd formula, we classified execution time results in three categories: below average (>12.7 s), average (11.7–12.7 s) and above average performance (<11.7 s). With this classification, the average performance covers a range of one second and falls into the middle 40 percent of total (from 30 to 70 percentiles). Results above 13 s are considered as very low (correspond to about 10th percentile), and results below 11.2 s as very high (correspond to about 90th percentile).

### 3.3. Shooting Efficiency Results

The players showed relatively low shooting performance with high variability (Table 2). Only one player achieved a goal from both the 5 and 7 m lines during the first attempt. In the second attempt, six players failed to score a goal regardless of the distance. Most of the players (44.4%) had a 25% shooting accuracy, five players (27.8%) had 50% and three players (16.7%) had 75% shooting accuracy, whereas two players (11.1%) had no successful shots. There was no player with all shots being successful. There was no difference in shooting accuracy between 7 m and 5 m shots (χ^2^(3,18) = 0.50; *p* > 0.05); however, shots from 5 m were about 1.4 times more likely to be successful than from 7 m.

**Figure 3:**
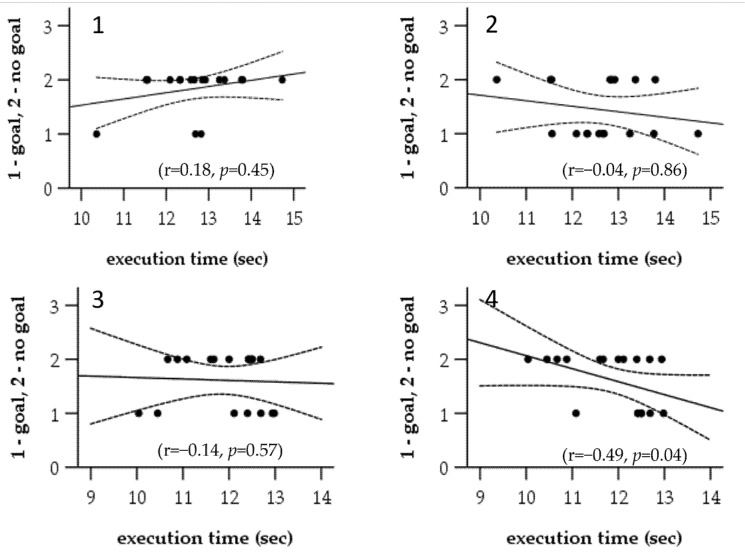
Results of the Spearman rank correlation analysis between the completion time and shooting performance. Panel **1** shows the correlation results between Attempt 1 mean execution time and shooting performance from 7 m. Panel **2** shows the correlation results between Attempt 1 mean execution time and shooting performance from 5 m. Panel **3** shows the correlation results between Attempt 2 mean execution time and shooting performance from 7 m. Panel **4** shows the correlation results between Attempt 2 mean execution time and shooting performance from 5 m. Dotted black line represents the upper and lower 95% confidence intervals.

Since there was no considerable difference in the measured execution times between Evaluator A and Evaluator B, we included the mean execution time for both evaluators in the correlation analysis. We did not find a significant correlation between the execution time during the first attempt and shooting performance from either 7 m or 5 m (Figure 3). We also did not detect a significant correlation between the execution time of the second attempt and shooting performance from 7 m, but there was a moderate negative correlation between execution time at Attempt 2 and shooting performance from 5 m.

## 4. Discussion

The main goal of this study was to develop a test simulating offensive agility in water polo and to examine its reliability between executions. An important aspect in the development of this functional test was to mimic typical offensive moves such as: high-intensity short-term direction changes and manoeuvering, body position changes (from horizontal to vertical and oppositely), having open skill characteristics with quick decision-making actions and also including a shooting task after perceiving an unknown stimulus. The main finding was that the test showed good reliability results between attempts and between evaluators.

Based on the literature, agility includes two subcomponents, change of direction speed and perceptual and decision-making factors [26]. In addition, it has been emphasized that sport-specific tests should rely on different cognitive skills to enhance and maximize the players’ performance. Morral-Yepes et al. [27] have suggested that tests including human movements as signals show a more ‘reality-like’ and sport-specific stimulus.

The mean execution time in this study (12.26 s) was close to the average active offense time in water polo gameplay [28], confirming that the temporal characteristics of this test well represent a real offensive situation. The test we used in this study was different in many aspects from those that were applied in water polo; thus, it is difficult to compare these tests with ours.

Measurements by Evaluator A and B were in acceptable agreement and showed good intrarater reliability (Figure 2), which confirms our main assumption, indicating that this test is reliable and in good alignment with the recommendation of Sheppard et al. [7]. Physical tests that can produce 0.8 or higher ICC values and CV values less than 8% are considered reliable tests [25]. The differences between Evaluators were small at both measurements and were higher than those reported by Tucher et al. [15] (ICC = 0.97, 0.98 vs. ICC = 0.88, respectively). To our knowledge, the study of Tucher et al. [15] is the only study in water polo that reported results with measurements by two evaluators.

We demonstrated higher reliability between executions compared to Dong et al. [1], (Stop and Go agility test ICC = 0.79; Jump and Go agility test ICC = 0.82), but it must be noted that in their study, the age group was significantly younger, and the test was based on reacting to light visual stimuli. Our results showed similar reliability to the agility test presented by Tucher et al. [15] (ICC = 0.87 between repetitions), in which the defensive actions of water polo were mimicked and the test duration was much shorter. In addition, the reliability of this study showed similar results to others who previously reported reliability results in agility tests conducted in team sports [6,10,29]. The complexity of this test and the aquatic environment add a natural variability to the performance; this factor should be considered when interpreting the results.

Relatively few studies have examined shooting skills in water polo [23,24,30]. In these studies, throwing efficiency and its connections with different features of water polo (e.g., the effect of fatigue on shooting velocity, accuracy, etc.) were measured, but the open skill aspects were not considered in a simulated attacking gameplay situation. Stevens et al. [24] found no differences in accuracy between shooting from a stationary position and after sprint swimming. The participants in that study were female players and the authors explained their findings with the initial low accuracy already from the stationary position leaving no room for further decrease [24]. Our study included more visual stimuli by the tester players, and the swimming consisted of intensive manoeuvres between the shots. In addition, the two separate shot attempts were required to be executed as quickly as possible (before and between the shots, there was a quick manoeuvre followed by signal recognition); thus, it imposed a greater time pressure on the players. Overall shooting accuracy from both sites (7 m and 5 m) (37.5%) is comparable with reports from official matches, where shooting accuracy was 39.9% for winner teams in male players [31]. Interestingly, accuracy did not differ between the 7 m and 5 m conditions. We initially expected significantly higher accuracy for shots from the 5 m line.

The correlation analysis demonstrated a lack of connection between shooting performance and execution time. Players were asked to perform the agility test as quickly as possible, which assumes maximal intensity. These results indicate that accuracy is unaffected by execution time as far as intensity is close to maximal, even with differences between players of three to four seconds. The complexity of the applied protocol should also be considered, since execution time depended not only on swimming and moving speed, but also to a certain extent on individual cognitive skills responsible for fast processing of the given stimuli. This was not directly measured in our study, but it may have influenced the association of execution time with shooting accuracy. Our results are in contrast with those reported by Platanou and Botonis [30], who concluded that better swimming performance would result in greater shooting accuracy, whereas an inefficient swimming technique seems to decrease shooting accuracy. This discrepancy may be because in the abovementioned study, the shooting accuracy and swimming performance were measured in a closed skill nature, and the evaluated players were not affected by any unknown stimulus.

This study has some methodological limitations that must be addressed. The sample size used in this study was small to allow for any generalizations and included only young athletes. Although such a sample size is common in similar reliability studies [32,33,34,35], this is a considerable limitation. Therefore, the results and the performance evaluation are limited only to players of similar age and cannot be transferred to players of different training background. We examined the reliability of this functional test, but not its validity and its discriminating power according to the competitive level of the players (elite vs. non-elite). Future studies should include a larger sample size with players of different age and competitive level. We did not measure the fitness level and swimming technique of the participating water polo players, which can influence their performance. In addition, the participants were not grouped by playing position since anthropometric differences can discriminate among positional requirements in water polo [11]. Decision-making elements were limited to two possible choices (left or right) similar to Sekulic et al. [6], but it is believed that future studies should focus on including multiple reaction possibilities [4] and/or integrate other types of stimuli, e.g., light stimuli behind the gate in the Sniper equipment.

## 5. Conclusions

The goal of this study was to develop a test containing offensive elements of water polo. The novelty of the test is that the athlete should move as quickly as possible in accordance with a random signal made by a tester player while having to throw at a goal from 7 m and then from 5 m. The protocol presented in this study was found to be a reliable measurement tool for testing offensive agility performance based on the open skill nature of water polo.

## Figures and Tables

**Table 1:** Results of the agility test measurements recorded by Evaluators A and B. Mean, standard deviation (SD) and coefficient of variation (CV).

	Evaluator A			Evaluator B		
	Attempt 1	Attempt 2	Mean	Attempt 1	Attempt 2	Mean
Mean (s)	12.64	11.93	12.29	12.59	11.85	12.22
SD	1.02	0.88	0.95	1.02	0.95	0.99
CV (%)	8.10	7.39	7.76	8.12	8.05	8.09

**Table 2:** Results of the attempted shots from 7 and 5 m during the two attempts. X represents the failed attempt and 0 represents the successful attempt.

Player	Attempt 1		Attempt 2		7 m Shooting Accuracy (%)	5 m Shooting Accuracy (%)	Shooting Accuracy (%)
	7 m	5 m	7 m	5 m			
1	X	0	X	X	0	50	25
2	X	X	0	X	50	0	25
3	0	0	X	0	50	100	75
4	X	0	X	X	0	50	25
5	X	X	X	0	0	50	25
6	X	0	0	X	50	50	50
7	X	X	X	X	0	0	0
8	X	X	X	X	0	0	0
9	X	0	X	X	0	50	25
10	0	X	X	X	50	0	25
11	X	0	X	0	0	100	50
12	X	0	0	X	50	50	50
13	X	0	X	0	0	100	50
14	X	0	0	0	50	100	75
15	0	X	0	X	100	0	50
16	X	0	0	0	50	100	75
17	X	X	0	X	50	0	25
18	X	X	X	0	0	50	25
Summary	18/3	18/10	18/7	18/7	27.8	47.2	37.5

## Data Availability

Data supporting the reported results in this study are available upon request from the corresponding author.
